# Clinical and immunological characterization of a Netherton syndrome infant with a large SPINK gene cluster deletion and a c.1258A>G polymorphism in *SPINK5*

**DOI:** 10.3389/fimmu.2025.1658444

**Published:** 2025-09-25

**Authors:** Yaning Guan, Qian Li, Yongjing Liu, Pingping Zhang, Maolin Huang, Yimin Guo, Jing Chen, Yan Chen, Zuochen Du, Pei Huang

**Affiliations:** ^1^ Department of Pediatrics, Affiliated Hospital of Zunyi Medical University, Zunyi, China; ^2^ Guizhou Children’s Hospital, Zunyi, China; ^3^ Laboratory of Hematology and Immunology, Guizhou Children’s Hospital, Zunyi, China

**Keywords:** SPINK5, Netherton syndrome, IVIG, immune modulation, immunophenotyping

## Abstract

**Introduction:**

Netherton syndrome (NS) is a rare autosomal recessive disorder caused by mutations in the *SPINK5* gene, which encodes the serine protease inhibitor LEKTI. It is characterized by congenital ichthyosis, hair shaft abnormalities, and atopic manifestations. Previous reports suggest that intravenous immunoglobulin (IVIG) may provide partial clinical benefit in NS. Here, we report the clinical and immunological characterization of an infant with NS effectively treated with IVIG therapy.

**Methods:**

Clinical information was collected and reviewed from a 1-year-6-month-old boy presenting with NS. Hair shaft abnormalities were examined by scanning electron microscopy. Pathogenic variants in *SPINK5* were identified using whole-exome and Sanger sequencing. Protein expression was assessed by Western blotting and ELISA. Peripheral lymphocyte subsets were analyzed by flow cytometry, and cytokine levels were evaluated with the Olink® Target 48 Cytokine panel.

**Results:**

The patient presented with typical clinical manifestations of NS. Genetic analysis identified a novel heterozygous deletion spanning the *SPINK5* gene (chr5:147,443,561-147,719,327), together with the c.1258A>G (p.K420E) polymorphism. LEKTI expression was markedly decreased, consistent with the genetic findings. Immune profiling revealed markedly reduced unswitched memory and marginal zone-like B cells, increased naïve B cells, and elevated γδ T cells compared with healthy controls. Cytokine analysis showed significantly increased levels of multiple pro-inflammatory cytokines, including TGFA, IL17 family members, CXCL8, CCL2, TNF, CCL19, and IL18. Following IVIG therapy, the patient demonstrated significant clinical improvement, with recovery of skin manifestations, and partial normalization of lymphocyte subsets and cytokine levels, indicating restoration of immune regulation.

**Discussion:**

This study reports a novel compound heterozygous *SPINK5* mutation in an infant with NS, comprising a large deletion and c.1258A>G polymorphism, resulting in LEKTI deficiency and immune dysregulation. IVIG therapy effectively alleviated clinical symptoms and restored immune balance, highlighting its potential as a therapeutic option for NS and related immunodeficiency disorders.

## Introduction

Netherton syndrome (NS) is a rare genetic disorder characterized clinically by a triad of ichthyosis, hair shaft abnormalities, and atopic manifestations (1). This condition results from mutations in the serine peptidase inhibitor Kazal type 5 (*SPINK5)* gene, encoding the serine protease inhibitor LEKTI (Lympho-Epithelial Kazal-type-related Inhibitor) (2). LEKTI is essential for maintaining skin barrier integrity and modulating immune responses and physiological desquamation by specifically inhibiting serine proteases kallikrein-related peptidase 5 (KLK5) and KLK7, among others (3). Mutations in *SPINK5* cause LEKTI deficiency, compromising the skin barrier function and increasing susceptibility to allergens and infections (4, 5). Clinically, NS typically manifests from birth with severe dermatological symptoms, including ichthyosis linearis circumflexa, erythroderma, and atopic-like dermatitis. These symptoms are often complicated by recurrent infections, growth retardation, and a variety of immunological abnormalities (6). Clinical management of NS is challenging, given the significant discomfort and diminished quality of life due to persistent dermatologic symptoms and related complications (7).

Recent studies have documented a diverse spectrum of *SPINK5* gene mutations associated with NS, including point mutations, small insertions, deletions, and splice-site mutations. Such mutations typically impair LEKTI function, producing the characteristic clinical phenotype of the syndrome (8). Although a number of specific mutation sites have been reported, such as nonsense mutations (e.g., p.R790*, p.R218*) and missense mutations (e.g., p.T808I, p.D106N), polymorphisms and large deletions in NS are less commonly documented (8–10).

Beyond affecting skin barrier integrity, *SPINK5* mutations significantly impact immune function. Patients harboring these mutations frequently exhibit compromised humoral immunity, characterized by reduced populations of specific B cell subsets, such as memory B cells, transitional B cells, and plasmablasts. Such immunodeficiency contributes to increased susceptibility to recurrent infections and inflammatory conditions (11, 12). Furthermore, elevated immunoglobulin E (IgE) and eosinophil counts commonly observed in these patients indicate a heightened allergic response (13). Dysfunctional LEKTI also disrupts the regulation of cytokine production, leading to an imbalance between pro-inflammatory and anti-inflammatory cytokines. Notably, elevated levels of interleukin-17 (IL-17) family cytokines and tumor necrosis factor-alpha (TNF-α) are frequently reported in patients with *SPINK5* mutations, fueling chronic inflammation and immune dysregulation (14, 15). A comprehensive understanding of these immune alterations is essential for the development of targeted therapeutic strategies aimed at modulating immune responses and improving clinical outcomes in NS.

Currently, NS remains incurable, and existing treatments primarily offer symptomatic relief. Systemic therapies, including topical calcineurin inhibitors (16, 17) and biological agents such as ustekinumab (18), have demonstrated variable efficacy. Nevertheless, there remains a significant gap in effective and targeted treatments for this challenging disease.

In this study, we conducted a comprehensive evaluation of the clinical, genetic, and immunological profiles of a 1-year-6-month-old patient presenting with severe NS. We assessed the extensive flaking affecting the patient’s entire body from birth, confirmed the presence and characteristics of genetic mutations and deletions through genetic testing, and measured LEKTI and KLK5 expression to elucidate the pathological implications of these genetic alterations. We comprehensively examined the changes in lymphocyte subpopulations, fine immune typing and various cytokine levels before and after the treatment to assess the modulation of disease immune function by intravenous immunoglobulin (IVIG). Through these evaluations, this study aims to deepen the understanding of the molecular and immunological mechanisms underpinning NS and highlight the therapeutic potential of IVIG in managing this complex and debilitating disorder.

## Materials and methods

### Patient recruitment and ethical considerations

This study involved a pediatric patient, a 1-year-6-month-old East Asian boy. Clinical data and blood specimens were collected after obtaining informed consent from his legal guardians during the patient’s initial hospitalization. Additionally, fourteen demographically matched (by age, race, and ethnicity) healthy controls were recruited. The study was conducted in strict adherence to the ethical principles outlined in the Declaration of Helsinki and was approved by the Ethics Committee of the Affiliated Hospital of Zunyi Medical University.

### Genetic analysis

To identify the genetic basis of the patient’s condition, previous genetic sequencing results were reviewed, which had been performed by the Center for Genetic Diagnosis of Rare and inherited skin diseases (Guangdong, China).

To confirm the *SPINK5* gene variant, genomic DNA was extracted from peripheral blood samples using a commercial DNA extraction kit (DP304-02, TIANGEN, China). PCR amplification was then performed using primers flanking the mutation site (19) (forward primer: 5’-CAGGGTTAGGCACATCACATTC-3’; reverse primer: 5’-TAAGGAATGCACGTGTTCCCTG-3’) (Sangon Biotech, China), and the resulting PCR products were subsequently validated by Sanger sequencing (Sangon Biotech, China).

### Cytokine analysis

Changes in multiple cytokines before and after treatment were analyzed using the Olink Target 48 Cytokine panel (Olink, China). Blood samples were collected from both the patient and healthy controls, and plasma was isolated through centrifugation of whole blood samples. The separated plasma samples were then submitted to Olink for cytokine profiling according to the manufacturer’s protocols.

### Scanning electron microscopy of hair

Hair samples obtained from the patient were sent to LiLai Biomedicine (Chengdu, China) for scanning electron microscopy analysis to characterize hair shaft abnormalities.

### Isolation of PBMCs

Peripheral blood samples were collected into vacutainers containing sodium heparin. The samples were first centrifuged at 2,500 rpm for 5 minutes at room temperature to separate plasma, which was collected and stored for subsequent analyses. The remaining blood cells were gently diluted with PBS and carefully layered onto Ficoll Paque PREMIUM (LTS1077, TBDscience, China). PBMCs were isolated by centrifugation at 500 × g for 20 minutes at room temperature, after which the PBMC fraction was carefully collected for further experimental procedures.

### Flow cytometry

Peripheral lymphocyte profiles were analyzed in a single experiment using 50 μL of whole blood, as previously described (20). The following antibodies, all purchased from BioLegend (USA), were used for cell staining: PE–anti-CD21 (354904), Brilliant Violet 421–anti-IgM (314516), PerCP–anti-CD38 (303520), Brilliant Violet 510–anti-IgD (348220), PE/Cy7–anti-CD27 (302838), APC–anti-CD19 (302212), APC/Cy7–anti-CD31 (303120), AF488–anti-CD24 (311108), Brilliant Violet 711–anti-IgG (410740), APC/Cy7–anti-CD23 (338520), Percp–anti-CD3 (300326), FITC–anti-CD4 (300506), APC–anti-CXCR3 (353708), Brilliant Violet 510–anti-CD8a (301048), PE–anti-CD127 (351304), PE/Cy7–anti-CD45RA (304126), Brilliant Violet 421–anti-CD185 (356920), PB–anti-CD38 (356628), Brilliant Violet 605–anti-CD27 (302830), PE/Fire640–anti-CD196 (353449), Brilliant Violet 711–anti-CD45RO (304236), Brilliant Violet 785–anti-PD-1 (329930), PE/Dazzle594–anti-CD57 (359620), AF660–anti-TCR γδ (331240), Brilliant Violet 650–anti-CD25 (302634), APC/Fire810–anti-HLA-DR (307674), PE–anti-TCR aβ (306708), Brilliant Violet 421–anti-TCR γδ (331218), PerCP–anti-CD3 (300326), FITC–anti-CD4 (300506), APC–anti-CD27 (356410), APC–anti-CD19 (302212), PE–anti-CD24 (311106), AF488–anti-IgD (348216), and PB–anti-CD27 (302822), APC–anti-CD20 (302310) and Zombie NIR™ Fixable Viability Kit (423106). Samples were analyzed on a FACS Canto Plus flow cytometer (BD Biosciences, USA) or Cytek^®^ Aurora/Northern Lights™ (Cytek, USA), and data analysis was performed using FlowJo software. The results were summarized and compared to age-matched healthy controls.

### Western blotting

PBMCs were lysed in RIPA buffer supplemented with protease inhibitors (Servicebio, China). Protein extracts mixed with loading buffer were separated by 10% SDS-PAGE gel electrophoresis and subsequently transferred onto PVDF membranes (Millipore, Germany). Membranes were blocked in 5% skim milk and incubated overnight at 4°C with primary antibodies against LEKTI (29808-1-AP, Proteintech, USA), antibodies against KLK5 (38528, SAB, USA) and HRP-conjugated β-actin recombinant rabbit monoclonal antibody (ET1702-67, HUABIO, China). After incubation with HRP-linked secondary antibodies (RGAR001, Proteintech, China), membranes were washed in TBST and visualized using an enhanced chemiluminescence detection system (BIO-RAD, USA).

### Enzyme-linked immunosorbent assay

Plasma was collected from peripheral blood samples, and the level of KLK5 was measured using a Human KLK5 ELISA Kit (JM-6832H2, JINGMEI, China). The assay was performed according to the manufacturer’s instructions, and KLK5 concentrations were calculated by interpolation from the standard calibration curve.

### Statistical analysis

Statistical analyses were performed using SPSS 29.0 (IBM, USA) and GraphPad Prism 8 (GraphPad Software, Inc., San Diego, CA, USA). Comparisons between groups were assessed using the unpaired two-tailed Student’s t-test. Statistical significance was defined as a *P*-value less than 0.05.

## Results

### Clinical presentation and medical history

The patient, a boy aged 1 year and 6 months, is the first child of healthy, non-consanguineous parents, born following an uncomplicated pregnancy. Immediately after birth, he exhibited widespread dermatological symptoms, including ichthyosis, erythroderma, and atopic-like dermatitis, characterized by severe scaling, erythema, and pruritus. These skin manifestations significantly affected his comfort and quality of life. The patient also experienced recurrent respiratory infections and persistent diarrhea, necessitating multiple hospital admissions and frequent antibiotic therapy. Additionally, he exhibited failure to thrive.

**Figure 1:**
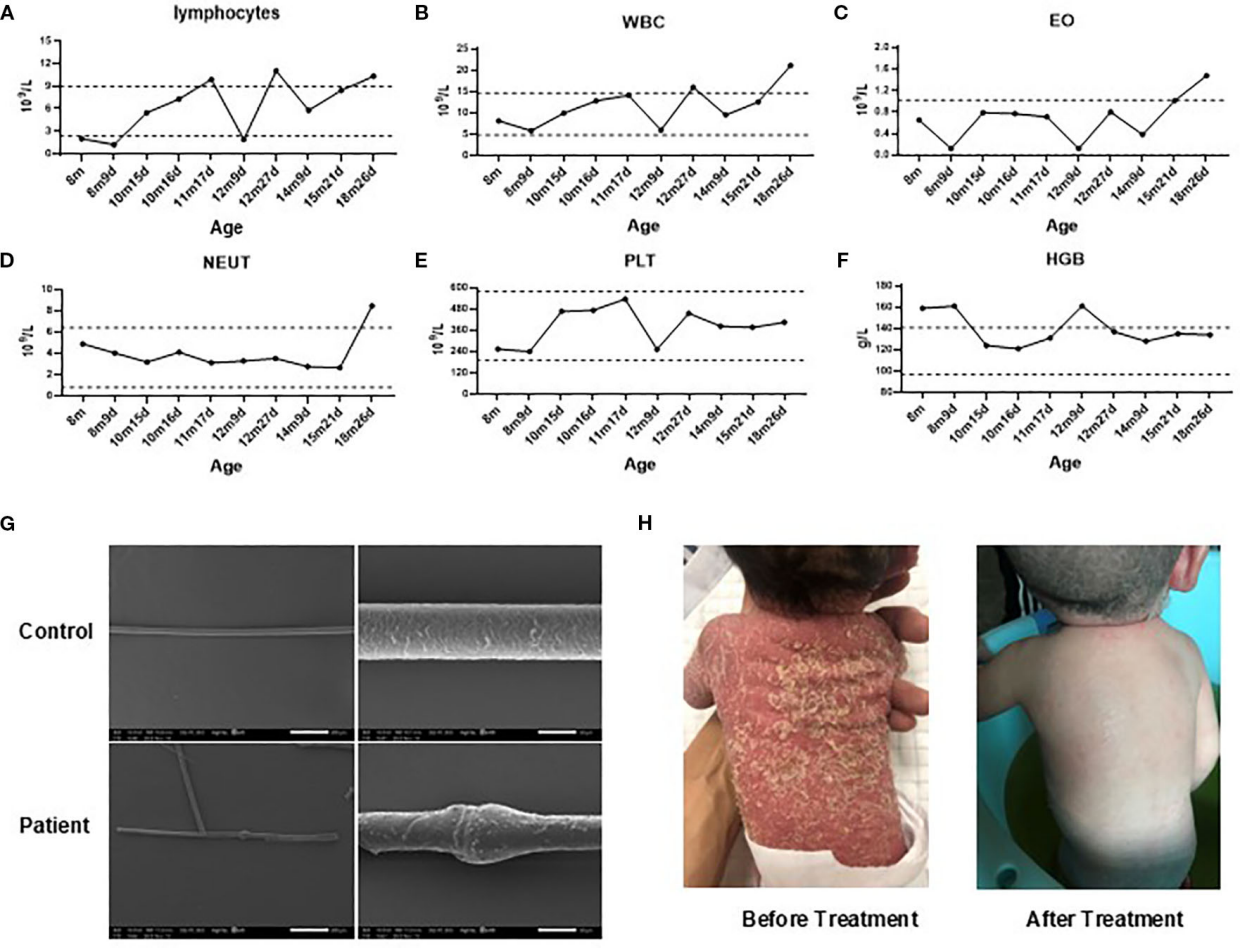
Clinical findings. **(A–F)**. Changes in the patient’s lymphocytes **(A)**, white blood cell counts (WBC, **B**), eosinophil counts (EO, **C**), neutrophils (NETU, **D**), platelet (PLT, **E**) counts and hemoglobin levels (HGB, **F**) over time. **(G)**. Scanning electron microscopy of hair from the patient and his mother. **(H)** Skin desquamation before and after IVIG treatment. Gray dashed lines represent reference ranges. m, months; d, days.

Routine blood examinations revealed that white blood cell (WBC) counts were generally within normal ranges, though elevations occurred during episodes of infection (**Figures 1A, B**). Consistently elevated eosinophil counts were noted (**Figure 1C**), whereas hemoglobin, platelet (PLT), and neutrophil levels remained within normal limits (**Figures 1D–F**). Scanning Electron Microscopy examination of hair shafts identified “bamboo hair” deformities (**Figure 1G**).

Since birth, the patient had received symptomatic skin care, including topical corticosteroids and emollients, along with specialized hypoallergenic formula feeding to manage potential food allergies. Despite these interventions, recurrent skin rashes and growth delays persisted. Regular IVIG therapy (500 mg/kg, once monthly) was initiated at 8 months of age. After 11 months of continuous treatment, the patient exhibited notable clinical improvement, including significant regression of skin lesions (**Figure 1H**), relief of gastrointestinal symptoms, and reduced frequency of respiratory infections. However, the family declined further therapy, which subsequently led to disease relapse.

### Genetic analysis and protein expression

**Figure 2:**
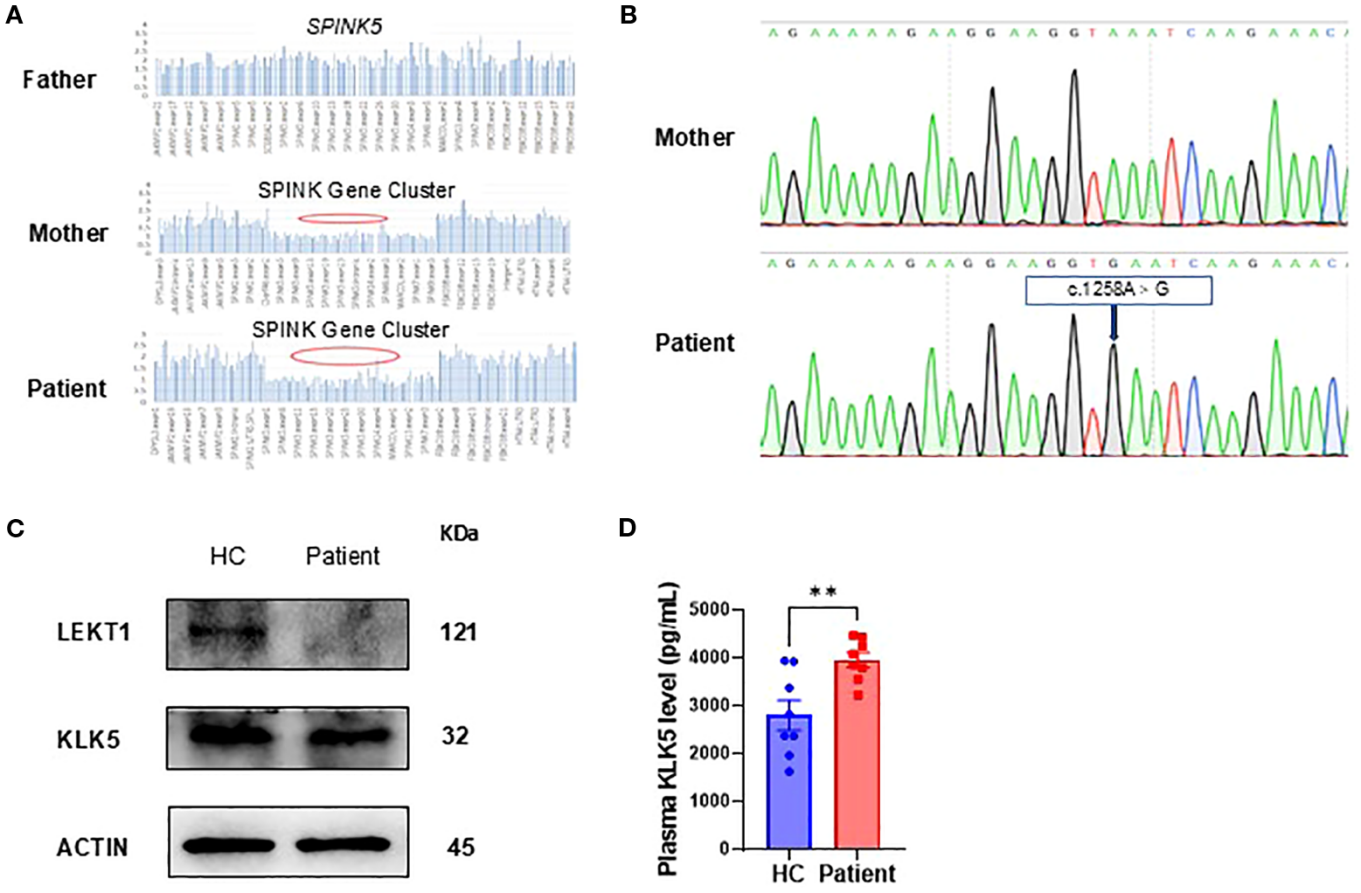
Genetic and protein expression analysis. **(A)** Whole-exome sequencing identified a heterozygous deletion spanning the SPINK gene cluster, including *SPINK5*. **(B)** Sanger sequencing of the patient and his mother revealed a polymorphism in *SPINK5* (NM_006846; exon 14, c.1258A>G, p.K420E). **(C)** LEKTI and KLK5 expression in peripheral blood mononuclear cells (PBMCs) from the patient compared to healthy controls (HC, n=3). **(D)** KLK5 level in the patient’s plasma (seven time points from initial hospitalization) compared with that in healthy controls (HC, n = 8).

We reviewed and validated the patient’s previous genetic testing results. Genetic analyses identified the presence of a G1258A polymorphism (NM_006846; exon14 c.1258A>G, p.K420E) and a heterozygous deletion of approximately 257.8 kb involving the *SPINK5* gene locus (**Figure 2A**). Sanger sequencing subsequently confirmed the G1258A polymorphism in *SPINK5* (**Figure 2B**).

To evaluate the functional consequences of these genetic alterations, we assessed LEKTI and KLK5 protein expression in PBMCs. The analysis revealed markedly decreased LEKTI and relatively low KLK5 expression in the patient compared with healthy controls, consistent with the identified genetic defects (**Figure 2C**). In contrast, we found a significant increase in plasma KLK5 levels (**Figure 2D**).

### Immunological assessment by multiparametric flow cytometry

We further investigated the patient’s immune function using multiparametric flow cytometry before and after IVIG therapy. Prior to IVIG administration, the patient’s B-cell compartments, including unswitched memory B cells, transitional B cells, and plasmablasts, were markedly reduced. Following IVIG therapy, these B-cell subsets significantly increased, suggesting an enhanced immune response and improved B-cell maturation. Although initial assessments of T-cell populations showed no notable abnormalities, post-treatment evaluations revealed substantial increases in total T cells, including CD8^+^ T cells, CD4^+^ T cells, CD4^+^ TEMRA cells, CD8^+^ TEMRA cells, CD4^+^ naive, and CD8^+^ naive T cells. These results indicate a substantial improvement in T-cell-mediated immunity following IVIG therapy (**Table 1**).

**Table 1:** Patient immunological profile.

Lymphocytes	8 months				1 year and 7 months			
	Percentage	Reference range	Number/μL	Reference range	Percentage	Reference range	Number/μL	Reference range
T cells	55.9 (L%)	55.32-73.11	3052.14	2187-6352	62.8 (L%)	53.88-72.87	6493.5	1794-4247
CD8^+^ T cells	18 (L%)	15.88-31.48	982.8	686-2278	29.4 (L%)	19.00-32.51	3040.0	580-1735
CD8^+^ naïve	93.2 (CD8%)	47.36-92.45	917.28	535-1677	48.1 (CD8%)	36.80-83.16	1457.9	356-1095
CD8^+^ TEMRA	1.02 (CD8%)	0.15-28.32	9.83	2-430	45.7 (CD8%)	0.84-33.02	1385.6	9-440
CD8^+^ CM	5.43 (CD8%)	4.82-24.11	53.51	51-316	3.4 (CD8%)	5.18-31.66	103.4	56-406
CD8^+^ EM	0.36 (CD8%)	0.2-8.94	3.55	2-120	2.7 (CD8%)	0.70-11.22	81.7	6-145
CD4^+^ T cells	33.2 (L%)	28.17-47.74	1812.72	1125-3768	28.1 (L%)	24.08-42.52	2905.5	902-2253
CD4^+^ naïve	75.3 (CD4%)	59.28-88.09	1365	764-2972	75.2 (CD4%)	46.14-84.40	2181.7	472-1760
CD4^+^ TEMRA	0.075 (CD4%)	0-1.49	1.37	0-33	1.7 (CD4%)	0.00-1.36	50.7	0-22
CD4^+^ CM	20.5 (CD4%)	10.15-33.38	371.83	206-796	20.4 (CD4%)	13.88-48.12	593.5	212-735
CD4^+^ EM	4.05 (CD4%)	0.42-3.96	73.71	11-60	2.7 (CD4%)	0.94-6.46	79.6	15-87
TCRαβ^+^ DNT	0.08 (T%)	0.41-1.55	2.43	16-58	0.5 (T%)	0.37-1.80	35.2	9-57
γδT	8.4 (T%)	3.95-10.40	256.62	128-436	7.7 (T%)	4.94-17.98	496.3	114-539
B cells	26.8 (L%)	17.20-29.71	1463.28	916-1832	13.9 (L%)	13.23-26.39	1437.3	461-1456
Unswitched memory B	0.11 (B%)	1.77-7.06	1.58	22-103	3.1 (B%)	2.98-14.18	44.5	26-124
Naïve B	97.2 (B%)	75.28-92.77	1419.6	726-1626	85.2 (B%)	65.54-86.62	1220.1	323-1089
Transitional B	5.81 (B%)	6.04-21.62	85.18	65-288	9.3 (B%)	5.24-17.22	133.4	35-172
Plasmablast B	0.095 (B%)	0.71-5.88	1.42	9-72	1.7 (B%)	0.50-7.06	24.8	4-63
NK cells	–	5.67-15.90	–	306-896	-	7.21-20.90	–	270-1053
CD4^+^:CD8^+^	1.84	0.93-2.52	–		1.0	0.90-2.13	–	

NA: Not Applicable."L%" means "percentage of lymphocytes". “CD8%” means "percentage of CD8^+^ T cells". “CD4%” means "percentage of CD4^+^ T cells". “T%” means "percentage of CD3^+^ T cells". B%” means "percentage of CD19^+^ B cells".Naïve: cytotoxic T lymphocyte with differentiation markers: CD3^+^ CD8^+^ CD45RA^+^ CD27^+^TEMRA: terminally differentiated effector memory: CD3^+^ CD8^+^ CD45RA^+^ CD27^−^CD8^+^ CM: central memory: CD3^+^ CD8^+^ CD45RA^−^ CD27^+^CD8^+^ EM: effector memory: CD3^+^ CD8^+^ CD45RA^−^ CD27^−^Naïve: helper T lymphocyte markers: CD3^+^ CD4^+^ CD45RA^+^ CD27^+^TEMRA: terminal effector memory differentiation: CD3^+^ CD4^+^ CD45RA^+^ CD27^−^CD4^+^ CM: central memory: CD3^+^ CD4^+^ CD45RA^−^ CD27^+^CD4^+^ EM: effector memory: CD3^+^ CD4^+^ CD45RA^−^ CD27^−^TCRαβ^+^ DNT: double negative T lymphocytes: CD3^+^ TCRαβ^+^ CD4^−^ CD8^−^Unswitched memory B cells: CD19^+^ CD27^+^ IgD^+^Naive B cells: CD19^+^ CD27^−^ IgD^+^Transitional B cells: CD19^+^ CD24^++^ CD38^++^Plasmablasts: CD19^+^ CD24^−^ CD38^++^.

### Lymphocyte subset assessment

**Figure 3:**
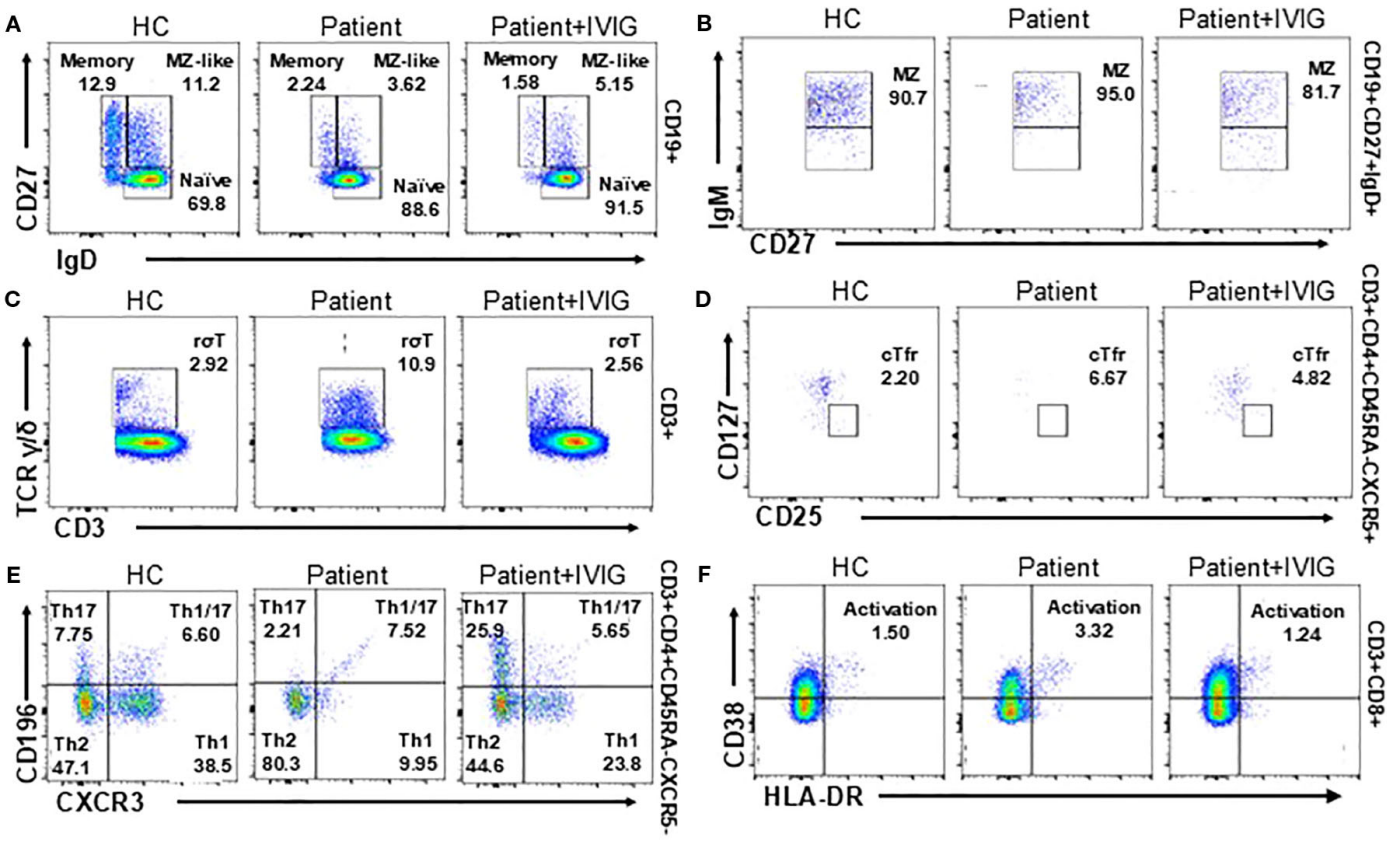
T and B cell subset phenotyping before and after treatment and comparison with healthy controls (HC, n=3). **(A, B)** Flow cytometry analysis of B cell subsets, including switched memory B cells, naive B cells **(A)** and CD27^+^IgD^+^ marginal zone-like (MZ-like) cells **(B)**. **(C-F)** Flow cytometry of T cell subsets, including γδ T cells **(C)**, cTfr cells **(D)**, Th cells **(E)** and activated CD8^+^ cells **(F)** in the flow cytometry.

To obtain a comprehensive view of the patient’s immunological status, we compared lymphocyte subsets before and after IVIG therapy with demographically matched healthy controls (HC). The patient exhibited significantly decreased proportions of switched memory B cells and CD27^+^IgD^+^ marginal zone-like (MZ-like) B cells compared with controls. Conversely, naive B cell populations were elevated in the patient, and this subset did not show significant changes following IVIG treatment (**Figures 3A, B**). Additionally, the patient showed markedly elevated proportions of γδ T cells compared to healthy controls; these were notably reduced following IVIG therapy (**Figure 3C**). The proportions of follicular regulatory T cells (cTfr), Th1/17 cells, and activated CD8^+^ T cells were significantly higher in the patient (**Figures 3D–F**). Post-IVIG therapy, these T cell subsets demonstrated a downward trend towards normalization.

### Cytokine profile analysis

**Figure 4:**
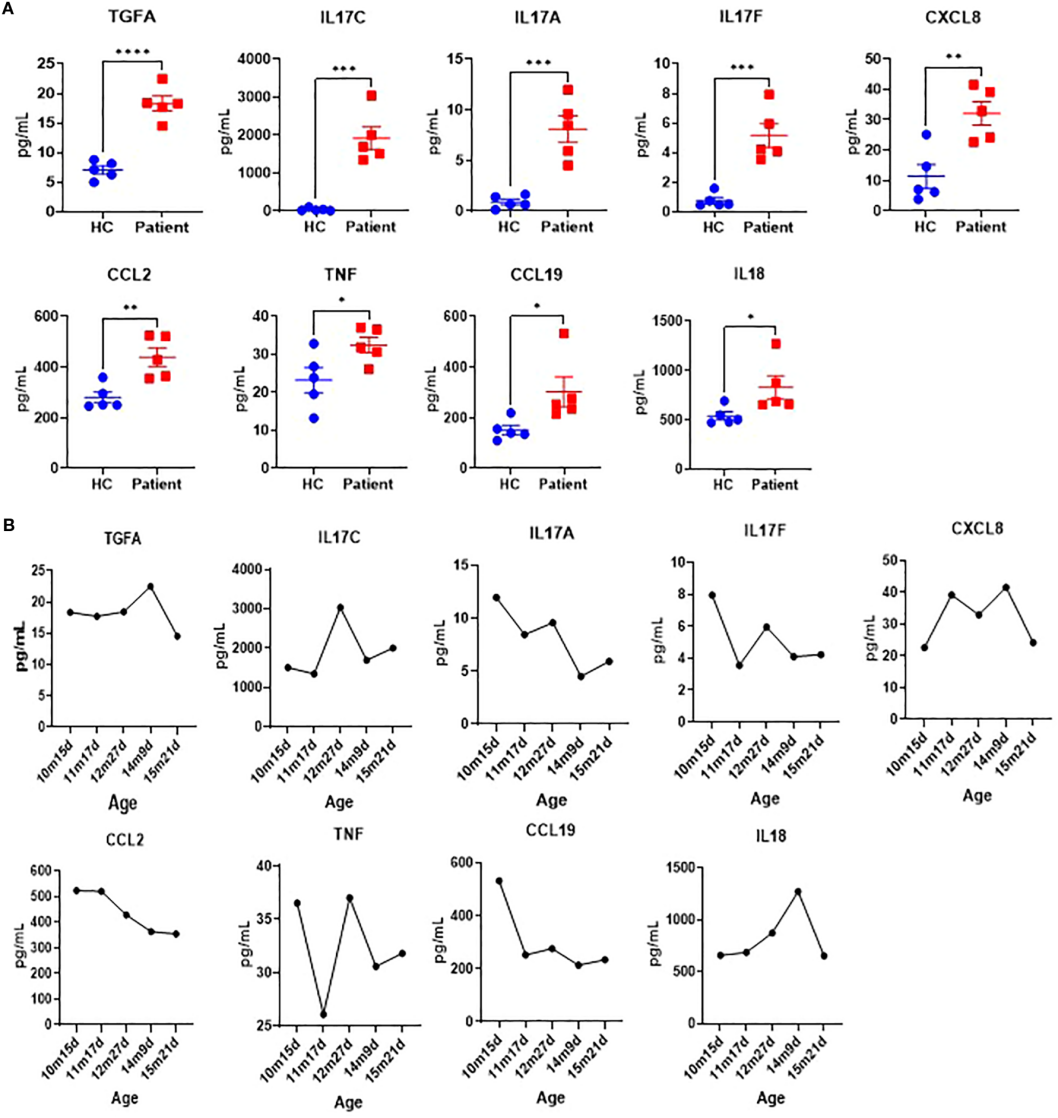
Cytokine profile analysis. **(A)** Cytokine profiles of the patient (measured monthly over five time points from initial hospitalization) and healthy control (HC, n=5). **(B)** Cytokine levels change before (10m15d) and after IVIG treatment over time.

To evaluate the immunological effects of the identified *SPINK5* mutation, a comprehensive multiplex cytokine profile analysis was performed. Our results revealed significant alterations in multiple key cytokines. Specifically, the patient exhibited notably elevated levels of TGFA, IL17C, IL17A, IL17F, CXCL8, CCL2, TNF, CCL19, and IL18 prior to IVIG therapy (**Figure 4A**). Following IVIG treatment, substantial reductions in the levels of CCL2, TNF, CCL19, IL18, IL17F, TGFA, and IL17A were observed (**Figure 4B**), indicating significant immune modulation by IVIG. These cytokines alterations likely contribute to the clinical improvements observed in the patient, such as reduced inflammation and enhanced immune regulation. This detailed cytokine profiling thus provides critical insights into the underlying therapeutic mechanisms of IVIG.

## Discussion

In this study, we conducted a comprehensive evaluation of a 1-year and 6-month-old pediatric patient with a confirmed diagnosis of NS, who presented with severe dermatological manifestations, immunological deficiencies, and growth retardation associated with mutations in the *SPINK5* gene. From birth, the patient exhibited hallmark clinical features, including ichthyosis, erythroderma, and atopic dermatitis, along with recurrent respiratory infections and gastrointestinal disturbances. Genetic analysis revealed a rare combination of a G1258A polymorphism and a heterozygous deletion spanning approximately 257.8 kb that encompasses the *SPINK5* gene. To date, only two cases involving large genomic deletions including the *SPINK5* gene have been reported worldwide (9, 21). *SPINK5* polymorphisms have been shown to affect LEKTI function and contribute to the clinical variability of NS (22, 23). To the best of our knowledge, this is the first reported case of a compound mutation comprising both a large fragment deletion and the c.1258A>G polymorphism in *SPINK5*, suggesting a potentially unique pathogenic mechanism and further emphasizing the functional heterogeneity of LEKTI-related deficiency. This mutation profile was associated with markedly reduced expression of LEKTI protein, confirming its pathogenic impact. Immunological assessments demonstrated a significant reduction in specific B-cell subsets, while T-cell populations remained within the normal range. Following IVIG therapy, substantial improvement in both B-cell and T-cell subsets was observed, indicating a restoration of immune functionality. In addition, multiplex cytokine profiling revealed notable modulation of inflammatory mediators after treatment, supporting the therapeutic efficacy of IVIG in modulating immune responses in NS.

Pathogenic variants of *SPINK5* associated with NS have been identified across all functional domains of the gene. To date, more than 80 distinct variants have been reported, including point mutations, deletions, and polymorphisms (8). Among these, point mutations are the most frequently documented, while reports of large deletions and polymorphisms remain relatively rare (9, 21). In our case, we identified a G1258A polymorphism in combination with a heterozygous deletion spanning approximately 257.8 kb that encompasses the entire *SPINK5* gene. To our knowledge, this combined large-fragment deletion and G1258A polymorphism represents a novel genetic alteration, further expanding the mutational spectrum of *SPINK5* and providing new insights into the genetic pathogenesis of NS. In NS patients, epidermal homeostasis is severely disrupted (with characteristic structural defects), accompanied by dysregulated expression of KLK5, a critical epidermal protease. This contrasts paradoxically with the well-established essential roles of KLK5/KLK7 in murine NS models (24, 25), suggesting gaps in understanding human NS pathogenesis-particularly given inconsistent reports on KLK5/KLK7 activity in clinical cases (26, 27). Our study demonstrates significantly decreased LEKTI expression in patient PBMCs, alongside relatively low KLK5 expression but elevated plasma KLK5 levels, consistent with previously reported findings (28). These findings reinforce the LEKTI-KLK5-axis role in disease pathogenesis and highlight the need for further investigation into the precise roles of these proteases in human NS pathophysiology.

Our detailed analysis of the patient’s lymphocyte subsets revealed several noteworthy findings, some of which align with existing literature on *SPINK5* mutations, while others provide novel insights. Prior to IVIG therapy, the patient exhibited markedly reduced levels of key B-cell subsets, including memory B cells, transitional B cells, and plasmablasts. These findings are consistent with previous reports of impaired humoral immunity in *SPINK5*-related immunodeficiency (12, 29). Notably, we also observed an increased proportion of naive B cells, which remained elevated even after IVIG treatment. This persistent elevation contrasts with prior studies, which have not emphasized this feature (12), suggesting a potential marker of disrupted B-cell maturation that may be resistant to IVIG modulation. Additionally, our study identified significantly elevated γδ T cells before treatment, which decreased after IVIG administration. This dynamic response of γδ T cells is rarely reported in the context of *SPINK5* mutations and highlights a previously underrecognized component of the T-cell response to immunoglobulin therapy. Moreover, the patient demonstrated elevated levels of cTfr, Th1/17 cells, and activated CD8^+^ T cells, populations associated with immune activation and inflammation. These subsets were substantially reduced following IVIG treatment, consistent with its known immunomodulatory effects and capacity to dampen pro-inflammatory responses (11). Importantly, the detailed profiling of these specific lymphocyte subsets provides a deeper understanding of the immune dysregulation associated with *SPINK5* mutations and the immune-restorative potential of IVIG. While prior studies have broadly characterized immune abnormalities in Netherton syndrome, our findings contribute novel perspectives by delineating how distinct B and T cell subsets respond to treatment. These insights not only reinforce existing knowledge but also suggest new avenues for immunophenotypic monitoring and tailored therapeutic strategies in patients with *SPINK5*-related immunodeficiencies.

Our comprehensive multiplex cytokine analysis revealed significant alterations in multiple cytokines following IVIG therapy, extending beyond previous studies that primarily focused on elevated IL-17 levels in patients with *SPINK5* mutations. While the increased expression of IL-17A, IL-17C, and IL-17F is consistent with existing literature (30, 31), our study identified additional cytokines with notable changes, including TGFA, CXCL8, CCL2, TNF, CCL19, and TNFSF12. The elevated levels of CXCL8 and CCL2, chemokines critical for recruiting immune cells to sites of inflammation (32, 33), along with TNF, a key pro-inflammatory cytokine (34), reflect a heightened state of immune activation. Furthermore, the upregulation of TNFSF12, involved in immune regulation (35), and CCL19, which plays a role in lymphoid tissue organization (36), provides new insight into the complex immune dysregulation observed in *SPINK5* mutation-associated disease. Following IVIG therapy, the observed decline in these cytokines suggests a robust immunomodulatory effect, likely contributing to the clinical improvements noted in our patient. This expanded cytokine profile not only deepens our understanding of the inflammatory landscape in *SPINK5*-related disorders but also introduces novel candidate biomarkers for monitoring disease activity and treatment efficacy. These findings support the use of cytokine profiling as a valuable tool in guiding therapeutic strategies and advancing personalized care for patients with *SPINK5* mutations.

In conclusion, this study underscores the considerable clinical and immunological challenges associated with *SPINK5* mutations and highlights the therapeutic potential of IVIG in improving immune function and alleviating disease manifestations. The identification of a G1258A polymorphism in combination with a heterozygous 257.8 kb deletion encompassing the *SPINK5* gene represents a novel genetic finding. Furthermore, the comprehensive profiling of cytokine dynamics and lymphocyte subset alterations provides new insights into the immunopathogenesis of NS. Although this study is limited to a single case, the findings emphasize the importance of further research involving larger patient cohorts to validate and expand upon these observations, as well as long-term efficacy evaluations to better understand the sustained therapeutic impact of IVIG in NS. Ultimately, such efforts will advance the diagnosis, treatment, and overall management of *SPINK5*-related disorders, contributing to improved outcomes and quality of life for affected individuals.

## Data Availability

The original contributions presented in the study are included in the article/supplementary material. Further inquiries can be directed to the corresponding author/s.
